# Plaques sclérales séniles

**DOI:** 10.11604/pamj.2017.28.31.11941

**Published:** 2017-09-14

**Authors:** Mohamed Elyadari, Amina Berraho

**Affiliations:** 1 Service d’Ophtalmologie B Hôpital des Spécialités, Chu IBN Sina Rabat, Quartier Souissi 6220 Rabat, Maroc

**Keywords:** Sclère, plaques, séniles

## Image en médecine

Nous rapportons le cas d'une femme âgée de 75 ans, hypertendue sous traitement, pseudophake au niveau de l'œil droit, cataracte de l'œil gauche et chez qui l'examen ophtalmologique décèle de façon bilatérale et symétrique des zones de dépression sclérale de couleur grisâtre située en avant de l'insertion des deux muscles droits internes. La plaque sclérale est le résultat d'une dégénérescence de la sclère liée à l'âge et est habituellement asymptomatique. La zone sclérale touchée devient mince et légèrement déprimée. Les lésions sont décrites comme étant bilatérales, symétriques, bien définies et de forme ovoïde. Elles sont habituellement situées en avant de l'insertion des muscles droits internes.

**Figure 1:**
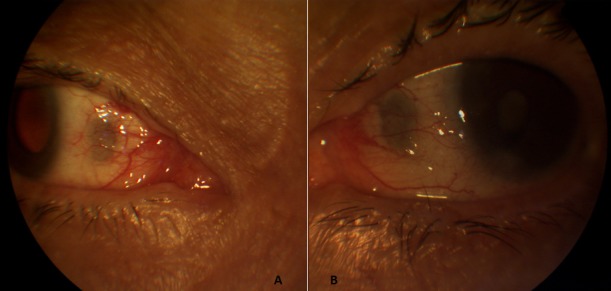
dépressions sclérales grisâtres ovoïdes situées en avant de l’insertion des deux droits médiaux: (A) œil droit; (B) œil gauche

